# Findings of Impaired Hearing in Patients With Nonfluent/Agrammatic Variant Primary Progressive Aphasia

**DOI:** 10.1001/jamaneurol.2018.4799

**Published:** 2019-02-11

**Authors:** Chris J. D. Hardy, Chris Frost, Harri Sivasathiaseelan, Jeremy C. S. Johnson, Jennifer L. Agustus, Rebecca L. Bond, Elia Benhamou, Lucy L. Russell, Charles R. Marshall, Jonathan D. Rohrer, Doris-Eva Bamiou, Jason D. Warren

**Affiliations:** 1 Dementia Research Centre, Department of Neurodegenerative Disease, University College London Queen Square Institute of Neurology, London, United Kingdom; 2 Department of Medical Statistics, Faculty of Epidemiology and Population Health, London School of Hygiene and Tropical Medicine, London, United Kingdom; 3 Preventive Neurology Unit, Wolfson Institute of Preventive Medicine, Queen Mary University of London, London, United Kingdom; 4 University College London Ear Institute and University College London Hospitals Biomedical Research Centre, National Institute for Health Research, London, United Kingdom

## Abstract

**Importance:**

Despite being characterized as a disorder of language production, nonfluent/agrammatic variant primary progressive aphasia (nfvPPA) is frequently associated with auditory symptoms. However, to our knowledge, peripheral auditory function has not been defined in this condition.

**Objective:**

To assess peripheral hearing function in individuals with nfvPPA compared with healthy older individuals and patients with Alzheimer disease (AD).

**Design, Setting, and Participants:**

This cross-sectional single-center study was conducted at the Dementia Research Centre of University College London between August 2015 and July 2018. A consecutive cohort of patients with nfvPPA and patients with AD were compared with healthy control participants. No participant had substantial otological or cerebrovascular disease; all eligible patients fulfilling diagnostic criteria and able to comply with audiometry were included.

**Main Outcomes and Measures:**

We measured mean threshold sound levels required to detect pure tones at frequencies of 500, 1000, 2000, 4000, and 6000 Hz in the left and right ears separately; these were used to generate better-ear mean and worse-ear mean composite hearing threshold scores and interaural difference scores for each participant. All analyses were adjusted for participant age.

**Results:**

We studied 19 patients with nfvPPA (9 female; mean [SD] age, 70.3 [9.0] years), 20 patients with AD (9 female; mean [SD] age, 69.4 [8.1] years) and 34 control participants (15 female; mean [SD] age, 66.7 [6.3] years). The patients with nfvPPA had significantly higher scores than control participants on better-ear mean scores (patients with nfvPPA: mean [SD], 36.3 [9.4] decibels [dB]; control participants: 28.9 [7.3] dB; age-adjusted difference, 5.7 [95% CI, 1.4-10.0] dB; *P* = .01) and worse-ear mean scores (patients with nfvPPA: 42.2 [11.5] dB; control participants: 31.7 [8.1] dB; age-adjusted difference, 8.5 [95% CI, 3.6-13.4] dB; *P* = .001). The patients with nfvPPA also had significantly higher better-ear mean scores than patients with AD (patients with AD: mean [SD] 31.1 [7.5] dB; age-adjusted difference, 4.8 [95% CI, 0.0-9.6] dB; *P* = .048) and worse-ear mean scores (patients with AD: mean [SD], 33.8 [8.2] dB; age-adjusted difference, 7.8 [95% CI, 2.4-13.2] dB; *P* = .005). The difference scores (worse-ear mean minus better-ear mean) were significantly higher in the patients with nfvPPA (mean [SD], 5.9 [5.2] dB) than control participants (mean [SD], 2.8 [2.2] dB; age-adjusted difference, 2.8 [95% CI, 0.9-4.7] dB; *P* = .004) and patients with AD (mean [SD], 2.8 [2.1] dB; age-adjusted difference, 3.0 [95% CI, 0.9-5.1] dB; *P* = .005).

**Conclusions and Relevance:**

In this study, patients with nfvPPA performed worse on pure-tone audiometry than healthy older individuals or patients with AD, and the difference was not attributable to age or general disease factors. Cases of nfvPPA were additionally associated with increased functional interaural audiometric asymmetry. These findings suggest conjoint peripheral afferent and more central regulatory auditory dysfunction in individuals with nfvPPA.

## Key Points

**Question:**

What is the status of peripheral hearing in patients with nonfluent/agrammatic variant primary progressive aphasia (nfvPPA)?

**Findings:**

Patients with nfvPPA performed worse on pure-tone audiometry than healthy older individuals or patients with Alzheimer disease, after controlling for age and general disease factors. In addition, these patients showed increased functional interaural audiometric asymmetry.

**Meaning:**

Auditory system involvement in patients with nfvPPA is more substantial than previously recognized.

## Teaser

This cross-sectional study compares peripheral hearing function in individuals with nonfluent/agrammatic variant primary progressive aphasia with that of patients with Alzheimer disease and healthy control participants.

## Introduction

Nonfluent/agrammatic variant primary progressive aphasia (nfvPPA) is characterized as a disorder of language production.^1^ Hearing in people with nfvPPA is not well characterized, but patients often experience difficulty following noisy and/or accented speech, word deafness, impaired recognition of voices and environmental sounds, and other symptoms potentially susceptible to auditory dysfunction.^1,2,3,4,5^ Besides its implications for the neurobiology and diagnosis of primary progressive aphasia, characterization of auditory dysfunction in patients with nfvPPA might help elucidate the potentiating role of hearing impairment in other neurodegenerative disorders (eg, Alzheimer disease [AD]).^2,6^ Here we assessed peripheral hearing using pure-tone audiometry in patients with nfvPPA compared with healthy control participants and patients with AD.

## Methods

### Participant Characteristics

We recruited patients with nfvPPA, patients with AD, and control participants between August 2015 and July 2018. All patients fulfilled diagnostic criteria,^7^ supported by neuropsychological assessment and brain magnetic resonance imaging (eMethods in the Supplement). No participant had a history of substantial otological disease or major comorbid cerebrovascular burden.

Ethical approval was granted by the University College London and National Hospital for Neurology and Neurosurgery research ethics committees. All participants gave informed consent consistent with Declaration of Helsinki guidelines.

### Audiometry Procedure

We adapted a standard clinical audiometry protocol^8^ assessing frequencies 500, 1000, 2000, 4000, and 6000 Hz (details in the Supplement). The participant’s mean threshold level for detecting each frequency was recorded in each ear.

### Data Analyses

Demographic and clinical characteristics were compared between groups using analysis of variance and Fisher exact tests. Audiometry data were analyzed by adapting a previously described protocol.^6^ For each participant, composite left-ear mean, right-ear mean, better-ear mean (BEM), and worse-ear mean (WEM) threshold and interaural difference scores were calculated across mean threshold levels for all frequencies. These scores were compared between groups using analysis of covariance models, with participant age as a covariate; pairwise group differences were assessed using planned comparisons that also adjusted for age. We conducted a separate analysis relaxing normality and homoscedasticity assumptions to check robustness (eTable in the Supplement). For descriptive purposes, we created categorical scores for each composite mean, with scores of 20 to 40 dB categorized as mild hearing loss, and scores greater than 41 dB categorized as moderate hearing loss.^8^

We used Spearman ρ to assess associations between age and both BEM and WEM scores across the entire cohort, and a series of partial correlation analyses (controlling for age) assessing associations between BEM and WEM scores and clinical duration, severity (via Mini-Mental State Examination [MMSE] score), and nonverbal executive function (Wechsler Abbreviated Scale of Intelligence [WASI] matrix reasoning score) in the combined patient cohort; and with measures of speech apraxia (polysyllabic word repetition score) and agrammatism (written sentence construction score) in the nfvPPA group. An α level of .05 was used as the statistical significance threshold. Data were analyzed with Stata version 14 (StataCorp).

## Results

Nineteen patients with nfvPPA, 20 patients with AD, and 34 control participants participated (Table 1). The control participants had a younger mean (SD) age (66.7 [6.3] years) than both patient groups (nfvPPA group, 70.3 [9.0]; AD group, 69.4 [8.1]), although the differences were not statistically significant (*P* = .19; Table 1). The inclusion of sexes was very similar across groups (control participants, 15 of 34 female [44%]; nfvPPA group, 9 of 19 female [47%]; AD group, 9 of 20 female [45%]; Fisher exact *P* > .99). There were no statistically significant differences between patient groups in mean (SD) symptom duration (AD group, 6.6 [4.0] years; nfvPPA group, 4.9 [2.1] years; *P* = .10), MMSE score (AD group, 18.6 [5.9]; nfvPPA group, 22.6 [7.1] points; *P* = .06), or executive function test result (AD group, 10.6 [6.6]; nfvPPA group, 13.9 [9.0]; *P* = .21).

**Table 1.** Demographic, Clinical, and Audiometric Characteristics of Participant Groups

Characteristic	Participants, No. (%)		
	Control Participants	Alzheimer Disease	nfvPPA
Total	34	20	19
Male	19 (56)	11 (55)	10 (53)
Age, mean (SD), y	66.7 (6.3)	69.4 (8.1)	70.3 (9.0)
Symptom duration, mean (SD), y	NA	6.6 (4.0)	4.9 (2.1)
Speech apraxia	NA	0	19 (100)
Expressive agrammatism	NA	0	12 (63)
Parkinsonian features	NA	0	11 (58)^a^
Supranuclear gaze palsy	NA	0	9 (47)^a^
Neuropsychological scores, mean (SD)			
Mini-Mental State Examination score^b^	NA	18.6 (5.9)	22.6 (7.1)
Wechsler Abbreviated Scale of Intelligence matrices^c^	26.1 (4.0)	10.6 (6.6)	13.9 (9.0)
Word repetition^d^	44.4 (1.2)	NA	33.8 (9.5)
Expressive agrammatism^e^	24.9 (0.4)	NA	17.5 (8.2)

Abbreviations: NA, not applicable; nfvPPA, patient group with nonfluent/agrammatic variant primary progressive aphasia.

^a^ Ten cases fulfilled current consensus diagnostic criteria suggesting underlying tauopathy: 9 with probable or definite progressive supranuclear palsy (Hoeglinger criteria) and 1 with probable corticobasal degeneration (Armstrong criteria). Of the remaining 9 patients with nfvPPA, 3 had confirmed pathological genetic mutations causing TDP-43 pathology (2 with progranulin and 1 with *C9orf72*). Hearing scores did not differ significantly between subgroups of patients with nfvPPA with and without probable or definite tauopathy (by analysis of covariance models adjusting for age: left-ear mean score, −3.4 [95% CI, −15.1 to 8.3]; *P* = .55; right-ear mean score, 5.9 [95% CI, −8.1 to 20.0]; *P* = .38; better-ear mean score, 3.5 [95% CI, −7.8 to 14.7]; *P* = .52; worse-ear mean score, 1.2 [95% CI, −13.0 to 15.3]; *P* = .86).

^b^ The Mini-Mental State Examination is on a scale of 30 points.

^c^ Wechsler Abbreviated Scale of Intelligence matrices data were missing for 1 control participant and 1 participant with AD; this test is on a scale of 32 points.

^d^ Word repetition data were missing for 4 control participants and 6 participants with nfvPPA; this test is on a scale of 45 points.

^e^ Expressive agrammatism data were missing for 4 control participants and 6 participants with nfvPPA; this test is on a scale of 25 points.

There was evidence of differences among the 3 groups in audiologic function in left-ear mean scores (control participants, 30.3 [7.2] dB; AD group, 32.7 [7.9] dB; nfvPPA group, 38.2 [10.2] dB; *P* = .02), right-ear mean scores (control participants, 30.4 [8.5] dB; AD group, 32.4 [8.0] dB; nfvPPA group, 39.6 [11.7] dB; *P* = .01), BEM scores (control participants, 28.9 [7.3] dB; AD group, 31.1 [7.5] dB; nfvPPA group, 36.3 [9.4] dB; *P* = .03), and WEM scores (control participants, 31.4 [8.1] dB; AD group, 33.8 [8.2] dB; nfvPPA group, 42.2 [11.5] dB; *P* = .002) (Table 2; Figure, A). Composite audiometric threshold scores were significantly higher in the nfvPPA group than control participants (age-adjusted differences: left-ear mean score, 6.3 [95% CI, 1.9-10.8] dB; *P* = .006; right-ear mean score, 7.2 [95% CI, 2.2-12.1] dB; *P* = .005; BEM score, 5.7 [95% CI, 1.4-10.0] dB; *P* = .01; WEM score, 8.5 [95% CI, 3.6-13.4] dB; *P* = .001) and in patients with AD (age-adjusted differences: left-ear mean score, 5.1 [95% CI, 0.2-10.0] dB; *P* = .04; right-ear mean score, 6.7 [95% CI, 1.2-12.1] dB; *P* = .02; BEM score, 4.8 [95% CI, 0.0-9.6] dB; *P* = .048; WEM score, 7.8 [95% CI, 2.4-13.2] dB; *P* = .005). There were no significant audiometric differences between the control participants and patients with AD (age-adjusted differences: left-ear mean score, 1.2 [95% CI, −3.1 to 5.6] dB; *P* = .57; right-ear mean score, 0.5 [95% CI, −4.3 to 5.4] dB; *P* = .83; BEM score, 0.9 [95% CI, −3.3 to 5.1] dB; *P* = .68; WEM score, 0.7 [95% CI, −4.0 to 5.5] dB; *P* = .77). Audiograms (Figure, B and C) showed elevated thresholds across frequencies in the nfvPPA group compared with the control and AD groups but a similar overall frequency sensitivity profile in all groups.

**Table 2.** Audiometry Results for Participant Groups^a^

Hearing Measurement Scores, Mean (SD), dB	Mean (SD)			Differences (95% CI)			*P* Value
	Control Participants	Participants With Alzheimer Disease	Participants With nfvPPA^b^	Participants With Alzheimer Disease vs Control Participants	Participants With nfvPPA vs Participants With Alzheimer Disease	Participants With nfvPPA vs Participants With Alzheimer Disease	
Left ear	30.3 (7.2)	32.7 (7.9)	38.2 (10.2)	1.2 (−3.1 to 5.6)	6.3 (1.9-10.8)	5.1 (0.2-10.0)	.02
Right ear^c^	30.4 (8.5)	32.4 (8.0)	39.6 (11.7)	0.5 (−4.3 to 5.4)	7.2 (2.2-12.1)	6.7 (1.2-12.1)	.01
Left-ear mean–right-ear mean difference^c^	−0.1 (3.6)	0.2 (3.6)	−0.7 (7.9)	0.5 (−2.3 to 3.4)	−0.2 (−3.1 to 2.8)	−0.7 (−4.0 to 2.6)	.90
Better ear	28.9 (7.3)	31.1 (7.5)	36.3 (9.4)	0.9 (−3.3 to 5.1)	5.7 (1.4-10.0)	4.8 (0.0-9.6)	.03
Worse ear^c^	31.7 (8.1)	33.8 (8.2)	42.2 (11.5)	0.7 (−4.0 to 5.5)	8.5 (3.6-13.4)	7.8 (2.4-13.2)	.002
Worse-ear mean–better-ear mean difference^c^	2.7 (2.2)	2.8 (2.1)	5.9 (5.2)	−0.2 (−2.0 to 1.7)	2.8 (1.0-4.7)	3.0 (0.9-5.1)	.006

Abbreviation: nfvPPA, patient group with nonfluent/agrammatic variant primary progressive aphasia.

^a^ Peripheral hearing composite scores for each participant were calculated by taking the mean threshold level required to hear tones at frequencies of 500, 1000, 2000, 4000, and 6000 Hz. Results from a separate analysis that relaxed normality and homoscedasticity assumptions and found similar results (eTable in the Supplement).

^b^ All values except the left-ear–right-ear mean difference score were significantly worse in participants with nfvPPA than both of the other participant groups.

^c^ Data for 1 participant with Alzheimer disease and 1 participant with nfvPPA were only available for the left ear.

**Figure.**
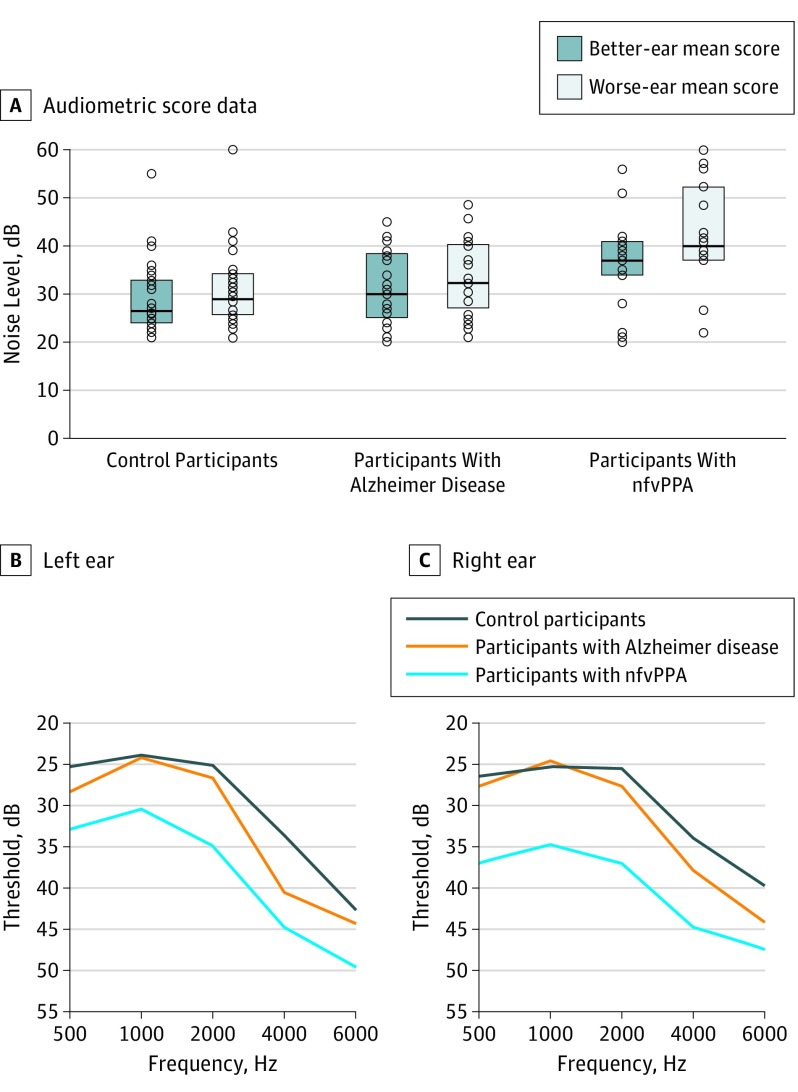
Pure-Tone Audiometry Scores Across Participant Groups Plots (A) show audiometric (composite ear and frequency score) data for individual participants within each diagnostic group. Boxes indicate interquartile ranges, and transverse lines indicate the median threshold for each participant group. Left ear (B) and right ear (C) audiograms show mean thresholds for detection of tones at frequencies of 500, 1000, 2000, 4000, and 6000 Hz for each participant group. nfvPPA indicates nonfluent/agrammatic variant primary progressive aphasia.

Of 34 control participants, 4 each had moderate hearing loss in the left ear (12%) and the right ear (12%). Of the 20 participants with AD, 5 had moderate hearing loss in the left ear (25%) and 3 in the right ear (16%). Of 19 patients with nfvPPA, moderate hearing loss was present in the left ear in 7 individuals (37%) and in the right ear in 7 individuals (39%).

There was also evidence of differences among the 3 groups in WEM-BEM difference scores (mean (SD): control group, 2.7 [2.2] dB; AD group, 2.8 [2.1] dB; nfvPPA group, 5.9 [5.2] dB; *P* = .006), these being significantly higher in the nfvPPA group than in control participants (age-adjusted difference, 2.8 [95% CI, 0.9-4.7] dB; *P* = .004) and patients with AD (3.0 [95% CI, 0.9-5.1] dB; *P* = .005). Worse-ear mean–better-ear mean (SD) difference scores were comparable in the AD and control groups (age-adjusted difference, −0.2 [95% CI, −2.0 to 1.7] dB; *P* = .85). There was no evidence of differences among groups in left-ear mean–right-ear mean (SD) difference scores (control group, −0.1 [3.6] dB; AD group, 0.2 [3.6] dB; nfvPPA group, −0.7 [7.9] dB; *P* = .90). Findings from the parallel analysis relaxing normality and homoscedasticity assumptions were substantially unchanged (eTable 1 in the Supplement).

Across the combined participant cohort, age was associated with WEM scores (ρ = 0.48; *P* < .001) and BEM scores (ρ = 0.46; *P* < .001). Across the patient cohort, after adjusting for age, there were no significant effects of disease duration, MMSE, WASI matrices, or language production scores on WEM scores (duration partial *r* = −0.18; *P* = .30; MMSE, partial *r* = 0.04; *P* = .82; WASI matrices, partial *r* = 0.08; *P* = .66; word repetition, partial *r* = −0.34; *P* = .30; sentence construction, partial *r* = −0.45; *P* = .17) or BEM scores (duration, partial *r* = −0.10; *P* = .56; MMSE, partial *r* = 0.10; *P* = .55; WASI matrices, partial *r* = 0.21; *P* = .23; word repetition, partial *r* = −0.12; *P* = .72; sentence construction, partial *r* = −0.32; *P* = .34).

## Discussion

In this analysis, we present evidence that patients with nfvPPA perform worse on pure-tone audiometry than healthy older individuals or patients with AD. These data suggest that this is not attributable to age or general disease factors. Moreover, nfvPPA was associated with increased interaural functional asymmetry, not lateralized to the right ear or left ear. The role of the auditory system in this language-led dementia has not been defined. These findings suggest that auditory pathway involvement in nfvPPA is more significant than generally recognized, corroborating the diverse hearing alterations (extending to environmental sounds and music) previously reported in these patients^1,2,3,4,5,9,10,11^ and proposed to contribute to the pathogenesis of nfvPPA.^4,10^

While this study has not defined the neural substrate for audiologic impairment in nfvPPA, there are several candidates. Impaired pure-tone audiometry usually signifies peripheral auditory dysfunction; most cases of nfvPPA are underpinned by tauopathy (encompassing corticobasal degeneration and progressive supranuclear palsy^1^; Table 1), and brainstem and subcortical pathways, including auditory pathways, are vulnerable to this pathology. However, involvement of peripheral auditory afferents in tauopathies does not necessarily produce audiologic deficits.^12^ Audiologic impairment in individuals with nfvPPA might additionally reflect involvement of cerebral integrative or brainstem efferent regulatory processes, such as those involved in auditory target detection.^13,14^ Any such dysregulatory effect would tend to be amplified in background noise, consistent with clinical observations.^1,2^ The finding of increased interaural functional asymmetry in individuals with nfvPPA is unlikely to be attributable to cochlear or auditory nerve pathology and implicates more central pathways, although its mechanism remains to be established.

Future work should address the mechanism of audiologic impairment in individuals with nfvPPA in association with cognitive and speech output functions in this syndrome and effects on patients’ daily lives. Auditory processing in these patients should be further characterized, both physiologically (including tympanometry, otoacoustic emissions, brainstem auditory evoked potentials, dichotic listening, and other central hearing tasks) and neuroanatomically (including structural and functional neuroimaging techniques) to establish the nature and locus of their hearing impairment.

## Conclusions

Consistent with recent work,^2,4,10^ these findings suggest that patients with nfvPPA have a disorder of communication signal processing that extends beyond neurolinguistic impairment; it may be timely to reevaluate the progressive aphasias from this fresh perspective. As a paradigm of selective neural system degeneration, nfvPPA could serve as a model disorder for interpreting the interplay of peripheral hearing and cognitive function in neurodegenerative disease and evaluating physiologically informed hearing and communication therapies in people with dementia. These could include interventions to improve the fidelity of auditory signal processing in noisy environments and harness residual plasticity in the damaged auditory system, an approach that shows early promise in patients with AD.^15^

## Notes

Supplement. **eMethods.** Details of audiometry procedure **eTable.** Comparisons of group audiometric data relaxing normality assumptions **eReferences.**
