# Supplementary material for: Findings of Impaired Hearing in Patients With Nonfluent/Agrammatic Variant Primary Progressive Aphasia
Source: JAMA Neurol. 2019 Feb 11;76(5):607–11. doi: 10.1001/jamaneurol.2018.4799 (PMC6515576; doi:10.1001/jamaneurol.2018.4799)
Supplement: Supplement. — eMethods. Details of audiometry procedure eTable. Comparisons of group audiometric data relaxing normality assumptions eReferences. [file jamaneurol-76-607-s001.pdf]

Supplementary Online Content

Hardy CJD, Frost C, Sivasathiaseelan H, et al. Findings of impaired hearing in patients with nonfluent/agrammatic variant primary progressive aphasia. *JAMA Neurol.* Published online February 11, 2019. doi:10.1001/jamaneurol.2018.4799

eMethods. Details of audiometry procedure

eTable. Comparisons of group audiometric data relaxing normality assumptions

eReferences.

This supplementary material has been provided by the authors to give readers additional information about their work.

© 2019 Hardy CJD et al. *JAMA Neurology.*

eMethods. Details of audiometry procedure

Using an Otovation Roto® audiometer (https://www.auditdata.com/) with a single TDH-39P 10-ohm Telephonics® earphone (www.telephonics.com) in a quiet room, steady tones of 500, 1000, 2000, 4000 and 6000Hz were presented separately to each ear, over ascending intensity levels commencing at 20dB HL (decibel hearing level). At each frequency, the participant indicated (verbally or by gesture) when they first heard a noise. If the participant was unable to hear the tone, the level was increased in 5dB increments (maximum 70dB HL). This procedure was repeated three times.

eTable. Comparisons of group audiometric data relaxing normality assumptions.

Hearing measure	**AD vs Controls**	**nfvPPA vs Controls**	**nfvPPA vs AD**	**p-value**
LEM (dB)	1.24 (-2.67, 5.11)	6.33 (1.29, 11.63)	5.09 (0.07, 10.46)	0.0185
REM (dB)[a]	0.52 (-3.71, 4.35)	7.18 (1.25, 12.95)	6.66 (1.03, 12.40)	0.0125
LEM-REM diff (dB)[a]	0.55 (-1.42, 2.42)	-0.16 (-3.84, 3.56)	-0.71 (-4.57, 3.20)	0.9008
BEM (dB)[a]	0.88 (-2.98, 4.63)	5.68 (0.73, 10.55)	4.80 (-0.00, 9.76)	0.0303
WEM (dB)[a]	0.70 (-3.45, 4.64)	8.51 (2.60, 14.19)	7.81 (2.14, 13.50)	0.0023
WEM-BEM diff (dB)[a]	-0.18 (-1.27, 0.95)	2.83 (0.74, 5.43)	3.01 (0.81, 5.68)	0.0051

[a]Data for one participant with AD and one participant with nfvPPA were only available for left ear. AD, patient group with typical Alzheimer's disease; BEM, better ear mean score; dB, decibels; diff, difference; LEM, left ear mean score; nfvPPA, patient group with nonfluent/agrammatic variant primary progressive aphasia; REM, right ear mean score; WEM, worse ear mean score. Peripheral hearing composite scores for each participant were calculated by taking the mean threshold level required to hear tones at frequencies of 500, 1000, 2000, 4000, and 6000 Hz.

The main manuscript reports results from parametric Analysis of Covariance (ANCOVA) models. In a separate analysis, we adopted a permutation approach attributable to Freedman & Lane[1]. This approach gives the same parameter estimates as ANCOVA but with p-values relaxing the normality and homoscedasticity assumptions made by ANCOVA; the p-values for the overall effect of diagnosis reported here were computed using 100000 permutations of residuals from age-adjusted models. We also calculated non-parametric bias corrected and accelerated bootstrap confidence intervals[2] for the between group differences based on 100000 bootstrap resamples; these also relax assumptions of normality and homoscedasticity. Results from this more conservative approach (tabulated here) were very similar to those using conventional ANCOVA models; albeit that the bootstrap confidence intervals for comparisons involving the nfvPPA group are typically (marginally) wider, and those for the AD vs Controls comparison are (marginally) narrower, than the parametric equivalents, but differences are all minor.

eReferences.

1. Freedman D, Lane D. A nonstochastic interpretation of reported significance levels. J Bus Econ Stat. 1983. doi: 10.1080/07350015.1983.10509354.
2. Efron B, Tibshirani RJ (1993). An introduction to the bootstrap, Chapman & Hall.

© 2019 Hardy CJD et al. *JAMA Neurology.*
